# Twelve Tips for Applicants from a Disadvantaged Background Considering a Career in Medicine

**DOI:** 10.15694/mep.2019.000144.1

**Published:** 2019-06-28

**Authors:** Awais Ul-Hassan, Salma Khanom, Penelope Sucharitkul, Christopher Mark Jones, Mohammed Abdul Waduud

**Affiliations:** 1 School of Medicine; 2 Stifford Tutoring; 3 Faculty of Biological Sciences; 4 Leeds Institute for Cardiovascular and Metabolic Medicine

**Keywords:** Disadvantaged Background, Medicine Application, United Kingdom, Tips, Advice, Widening Access.

## Abstract

This article was migrated. The article was marked as recommended.

A minority of medical school entrants draw from disadvantaged backgrounds, which remain significantly under-represented within the medical workforce. Whilst multifactorial, this may in part relate to relative lack of information about the admissions process amongst these groups. In this article, Mohammed Abdul Waduud and colleagues offer their twelve essential tips to support students from disadvantaged backgrounds who are considering applying to medical school. The authors, all of whom are from disadvantaged backgrounds, have experience in applying to medical schools within the United Kingdom. The tips within this article should support students from disadvantaged backgrounds to decide whether a career in medicine is right for them and succeed in their applications to study medicine.

## Introductions

In the United Kingdom (UK), despite a reported shortage of doctors, competition for medical school places remain rife. For every one place in medical school there are approximately eight applicants (Appendix 1) ( Medical Schools Council, 2019). However, it is estimated that only 4.1% of medical school entrants are from a disadvantaged background (British Medical Association). Furthermore, research by the Medical School Council highlights 80% of medical students to have studied at only 20% of the schools in the UK ( Garrud, 2014). Despite this, there is evidence to suggest that students from state schools outperform students with similar entrance grades achieved through the independent education system ( Espinoza, 2015). It is therefore unsurprising that medical schools are increasingly seeking to promote greater diversity and equality.

Several programmes relevant to this aim have been launched by medical schools throughout the UK. Of these, the widening access to medicine programmes are the most widely recognised ( Mathers *et al.*, 2011; Atkins and Ebdon, 2014; Dowell *et al.*, 2015; Cleland *et al.*, 2016; Steven *et al.*, 2016). The definition of candidates eligible for support from these programmes differs between medical schools; however, most agree that applicants must either have a: household income of £25,000 or less, be eligible for free school meals, be in the first generation of your immediate family to attend university, have disruption by a circumstance in their personal, social or domestic life, grew up or live in public care or live in a geographical area with low progression to higher education. This is by no means an exhaustive list (Appendix 2) ( Medical Schools Council, 2019). Furthermore, there is no evidence that any of these criteria impact on a person’s ability to practice medicine, yet students from these backgrounds remain underrepresented.

Despite the advent of these programmes, students from disadvantaged backgrounds continue to be poorly represented in medical school intakes. The causes for this are multifactorial and include differential attainment in school examinations and a relative lack of information and advice regarding the application process and the expectations for extra-curricular experience ( Kumwenda *et al.*, 2017). Given this, we provide here advice for students from disadvantaged backgrounds who are considering applying to medical school. These twelve tips should help students consider the realities of applying to study medicine, and to position themselves to succeed in the application process.

## Tip 1: Establish why you want to become a doctor.

This is arguably the most important part of the journey. The reason you want to study medicine is what forms the foundation of everything; it is what will push you through the application process and enable you to thrive. It is difficult for someone else to tell you what your motivation is as there is no right answer, so give it some careful consideration. Your motivation for studying medicine is something you must explore, define and elaborate. Being clear on this can be pivotal in determining the strength of your application, yet arguably it is often overlooked by applicants.

So why might you wish to study medicine? Many students will instinctively say: “I want to help people”. However, remember a career in medicine is a lifelong commitment and you may find it useful to reflect on how exactly you intend to help people. Think about the impact medicine has on patients. Is there anyone or a group of patients in particular you wish to help? Do you wish to take part in humanitarian work? The rationale for wanting to help people is endless. Alternatively, your reason for wanting to become a doctor may have sparked from a personal experience. Reflect on that. Or is it the undeniable satisfaction you believe doctors feel due to their ability to improve health? Discuss your motivation with family members, friends and teachers. They may have noticed certain qualities in you which you overlooked, and which may help you to clarify why you have chosen to study medicine.

## Tip 2: Talk to someone from the medical profession (if possible).

Speaking to medical professionals will give you invaluable first-hand insight into the career you are planning to pursue. They may also be able to answer questions you may have about the profession and the application process. Unfortunately, for those from disadvantaged backgrounds it can be challenging to gain one-to-one contact with a medical professional, not least because they may be the first in their family to pursue higher education. Nevertheless, a good place to start may be your family General Practitioner or practice nurse. Arrange a time to go into your GP practice and have a chat with them. Alternatively, you could simply have an opportunistic discussion with them during your next encounter. Most will be happy to help and offer advice.

Another avenue is to attend workshops and summer schools organised by medical schools. These are usually free to attend and are designed to give a broad insight into the life of medical students. Furthermore, it is a wonderful opportunity to speak and interact with them. Medical students will have all been through the application process relatively recently and can give specific advice about their respective universities’ application process.

## Tip 3: Read key texts to demonstrate that your decision to apply to study medicine is informed.

The organisation which oversees doctors, the General Medical Council (GMC), has authored two key publications which we would suggest all applicants be aware of. These are “Outcomes for Graduates” (General Medical Council) (previously known as “Tomorrow’s Doctors”) and “Good Medical Practice” (General Medical Council). These are aimed at medical students (especially final years) and detail the expectations of a doctor.

Outcomes for Graduates can be a laborious read but it is important to be aware it exists and you should try to familiarise yourself with the summary pages. Good Medical Practice is much shorter and is also important to read. Both resources describe qualities, characteristics and traits which are perceived to be important for a doctor. Furthermore, Good Medical Practice gives an accurate description of what will be expected of you during and after medical school. Applications will certainly stand out at any stage if either of these resources are mentioned.

## Tip 4: Try to do some work experience.

Work experience is often perceived as an integral part of the medical application. Any exposure which allows you to gain an appreciation of the healthcare profession will offer you a useful insight. For many applicants this will affirm their beliefs about becoming a doctor and it often forms the basis of their personal statement. Many students nevertheless struggle to find relevant work experience. However, don’t panic - with some persistence and patience something will come up.

Start by speaking to the careers lead at your sixth form or college as they may be able to give you valuable advice. Aim to secure work experience early and take advantage of whatever opportunity that is presented. The NHS offers work experience programmes lasting between 5-7 days within the hospital setting. However, waiting lists for these are often long therefore applying early is of paramount importance. To be in with the best chance of getting a place email the hospital as soon as possible. The earlier the better. You should be able to find a contact for this by typing the hospital name and “work experience” into Google. Introduce yourself, inform them you are applying for medicine and would appreciate it if they can offer you some work experience. If you are unable to get a place within the hospital setting, this is not the end of the world. Approaching a GP practice may be another avenue. Due to issues surrounding patient confidentiality you will not be permitted to undertake work experience at the practice you are registered with yourself. Try contacting other local practices instead and ask for the email of one of the GP partners or the practice manager.

Many students attending work experience do so as a means of ticking the box for their application. Some may believe that the location and duration of work experience will make them stand out from others. However, in reality being able to demonstrate what you have learnt from your experience is more important. Write a reflection about what you did and learnt each day so that when it comes to writing the personal statement and preparing for interviews you have something to refer to. In terms of the treatments patients receive, you will probably remember and understand very little. However, try to remember important characteristics medical professionals you shadowed demonstrated. These may include trust, communication, teamwork (multi-disciplinary teams), patient safety, ability to teach and life-long learning.

For more information and resources about work experience refer to *BMA Tips for Work Experience*:

https://www.bma.org.uk/advice/career/studying-medicine/becoming-a-doctor/work-experience-for-students .

## Tip 5: Volunteer for something or develop an extra-curricular passion.

Volunteering and pursuing extra-curricular activities support your personal development and are beneficial for your application; demonstrating your commitment and providing you with an opportunity to gain key skills. Taking part in schemes such as the Duke of Edinburgh (DofE) Award and/or National Citizen Service (NCS) will help formalise your participation in these activities and provide evidence of your commitment ( *The Duke of Edinburgh’s Award - The DofE in the UK*; National Citizen Service). However, remember the recipe for making the most of any experience is to take part, enjoy it, reflect upon it, apply it to medicine and be able to talk about it. Prices to take part in the DofE vary from £22-£29 depending on the level of the award you are aiming to complete, though there may be additional costs for equipment you require to complete the award ( *The Duke of Edinburgh’s Award - The DofE in the UK*). The NCS on the other hand, is free (National Citizen Service).

Whilst any volunteering experience will be welcome, you may be well served to spend your time in a caring environment that will provide you with additional skills and experience relevant to practicing as a doctor. Volunteering can include anything from mentoring at your school to volunteering abroad. Other examples include coaching sporting activities, working in a charity shop (e.g. British Heart Foundation, Cancer Research UK), working within a community group, serving teas in hospitals and care homes. You should demonstrate a genuine interest by taking part in a consistent manner, even if it’s for a short duration of time such as an hour a week.

You may already be doing an extra-curricular activity which may help exemplify the attributes you believe make a good doctor. Extra-curricular activities are varied and can include anything from musical instruments, programming or taking part in a competitive sport. Again, it’s completely up to you. Your school may offer these activities free or provide bursaries for disadvantaged students.

Many applicants seek to stand out from the crowd by doing several activities. However, keep in mind that this can also be counter-productive as the personal or specific qualities you are intending to exemplify may be overshadowed. So, spend some time planning and identifying what it is you want to demonstrate through the activity. Applicants who can demonstrate focused learning points may standout, especially when writing their personal statement and during interviews.

## Tip 6: Utilising the power of social media.

Social media is an excellent resource with an emerging presence in healthcare and is increasingly utilised by healthcare professionals. Most medical schools and affiliated institutions, medical students and doctors now have some sort of social media presence. Follow the medical schools you are interested in applying to. You will be kept updated on any events they are holding for applicants to attend, such as workshops, summer schools and mock interviews. It will also allow you to keep up to date with current affairs in medicine. You may also wish to follow feeds from BBC Health, the NHS or other reputable sources. This will allow you to have a greater appreciation of key medical issues within the NHS and further afield.

## Tip 7: The art of communication- make sure yours is up to scratch.

Throughout the entire application process your communication skills will be tested. Interviewers are often considering “Would I want this student as my doctor?”. If you are polite, confident and maintain good body language, this will give a hint to the interviewer about your future ‘bedside manner’.

The first thing to mention is unfortunately that you should be prepared to be ignored when contacting anyone for help/advice. Whilst this can be frustrating, be sure to maintain professionalism always. Carefully word all correspondence. Ask someone who is good at literacy to check over your communications. Remember to use a professional writing style, for example, Dear Dr. Smith and finish with Yours Sincerely (Name and School/College Year).

Once you’ve sent your correspondence if you don’t receive a reply within a couple of weeks don’t be afraid to send another reminder email. If there is a phone number available, give them a call. Organisations and individuals often receive many similar messages therefore persistence is key. However, do bear in mind that doctors have busy schedules and these may take precedence.

## Tip 8: Review the list of medical schools and identify your top four schools.

When applying for medicine, you can choose up to four medical schools as an undergraduate student. There is an additional fifth option which can be used to apply for a different course. Choosing may seem daunting but there are some key points to consider that will help you whittle your options down.

As your start point, you can readily find a list of UK medical schools online. The “Times Higher Education-The World University Rankings” and “The Complete University Guide” are two key sources for this (The Complete University Guide; Times Higher Education). It is a good idea to attend a couple of medical school open days to help refine your selection but it would be almost impossible to visit every medical school in the UK!

On key you should pay particular attention to is the entry requirements for each institution. Are you required to take the BMAT or the UKCAT? How well do you need to perform in these? Which A-Levels are needed, and what grades do they expect? How is the personal statement used in the application process? For example, the University of Birmingham do not score personal statements, whereas other medical schools such as the University of Exeter rank them ( Medical Schools Council, 2019). If anything is unclear or you have any questions, email or call the medical school to ask for clarification. Once again do this early so you have all the information you need to make your decision. A very useful resource to look into is “Entry Requirements for UK Medical Schools” produced by the Medical School Council ( Medical Schools Council, 2019).

Consider whether you want to live at home or live away (Medical School Council). The vast majority of students wish to live away from home, but a lot of students decide to commute from their family home every day ( Holton, 2018). It is important to be aware that living away from home incurs additional accommodation costs, which may be greater than those incurred by commuting. Depending on the household income of your parents, you can apply for an increased maintenance loan from Student Finance England to support your living costs (Student Finance England). Scholarships are definitely worth your time researching into, as you may not be directly informed about you qualifying for them- remember to chase this up early.

Choosing your fifth choice can be tricky. Put down a fifth choice if you are genuinely considering it as an alternative to medicine. It is completely fine to leave it blank especially if your alternative is to take a gap year to work on your personal statement. Common fifth choices include Biochemistry/Biomedicine (from which you may be able apply for post-graduate medicine), Optometry and Pharmacy.

Some medical schools may have a waiting list of applicants in the event preferred applicants miss the requirements or someone withdraws from the application process. You should not overly rely on such offers since sometimes they can result in disappointment. Some medical schools hold it as a condition that you have not been offered a place at any other university. For more information about this it is best to contact the medical school you are wishing to apply to.

## Tip 9: Choose the right subjects, but don’t worry if your grades aren’t outstanding.

Most medical schools will set a certain benchmark for grades. It is usually three grade As at A-level. Chemistry is usually a mandatory subject require and biology, physics and mathematics are some of the other preferred subjects. Requirements can vary ( Medical Schools Council, 2019).

If your grades do not meet this expectation don’t worry. The majority of medical schools have recently introduced access schemes whereby based upon an applicant’s background the minimum required grades may be reduced (Appendix 2) ( Medical Schools Council, 2019). For example, at the University of Leeds - there is a scheme known as “Access to Leeds” (University of Leeds). If eligible, your offer may be lowered by up to two grades. For example, from AAA to ABB. Many of these schemes have deadlines for applications, so make sure you identify these and apply for these schemes well in advance. A brilliant resource to have a look through and read is the BMA website on Widening Access (British Medical Association). If you open the tab “Useful Resource”, there are further places/links you can apply for funding for different courses which may help you with your medicine application.

Policies surrounding re-sitting examinations, like entry requirements, vary from university to university. Some medical schools accept A-Level results from resit examinations, e.g. Sheffield (University of Sheffield) and Lancaster (Lancaster University), whereas other schools do not, such as Birmingham (University of Birmingham) and Dundee (University of Dundee). We advise that if you are looking to apply for medicine but need to resit some exams, in order to meet the entry requirements, that you carefully research and think about the policies of the individual university you are thinking about applying too and identify the most up-to-date information (Medic Portal).

## Tip 10: Prepare for the exams.

All medical schools in the UK require applicants to sit the University Clinical Aptitude Test (UKCAT) and some require you to sit the Biomedical Admissions Test (BMAT). The content of these exams are described on multiple sources online (Medic Portal; Medic Portal). However in short, the UKCAT is a two hour computerised exam comprising of five distinct sections and can cost between £65-115 to sit. On the other hand the BMAT is also a two hour exam with a pen and paper comprising of three sections and costs £46. Develop an insight into what is expected well in advance.

There are several free online resources which may be used to prepare for the exams (Appendix 3). Alternatively, face-to-face courses may be attended which are run by external companies. It is important to emphasise that these are not essential to attend and most charge a fee. However, if there is a couple of courses that you are keen to attend it may be worth asking your school if they have any funding to help facilitate your attendance. This may in the form of a small scholarship or bursary. The BMA have enlisted a few other places where funding to attending these courses may be obtained (British Medical Association).

## Tip 11: Identify a few referees.

References are expected for most applications in life. Your application to medical school is no different. With the exception of family members the referee you choose can be anyone you have interacted with them on a professional level in the past. This could be a teacher, a mentor, the coach for a sporting activity you do or the manager from the place you volunteer or work. The list is by no means exhaustive. All they have to be able to do is support the claims reported in your application.

You should approach potential referees early. Discuss your ambitions and reasons for needing their support; brainstorm attributes you have demonstrated which will help you become the doctor you want to become.

## Tip 12: Finally have self-belief.

If you don’t believe in yourself no one else will. The authors of this article are a testament that it is possible to not only enter medical school if you’re from a disadvantaged background but thrive. Be prepared to utilise the application process to showcase your hard work and sell yourself to exemplify your aptitude for medicine.

Don’t ever be shy to demonstrate how hard you are working. However, try not to focus solely on your academic achievements. Try maintaining a healthy work-life balance by participating in other activities as aforementioned. Don’t be afraid, don’t be shy, and get ready to show admissions tutors why you deserve a place at their medical school.

## Conclusions

In conclusion, this article outlines our top tips which should be considered before applying to study medicine. Starting from a more disadvantaged background should not deter or prevent students and this article will help you start your journey. It is important to note that the key to success is early preparation, hard work, determination, and self-belief.

## Take Home Messages

• These twelve tips are aimed at encouraging disadvantaged students into medicine and offering advice from those who have achieved places in medical schools in the UK from disadvantaged backgrounds. Students are urged to consider each tip in order to maximise their chances of success.
• Careful consideration and planning of all aspects of your application will make you an appealing candidate to medical schools. Plans, prevent, poor, performance.
• Disadvantaged students can feel as though the odds are against them, with the already challenging application process fraught with additional barriers to prevent your entry into medicine. Be persistent and show how determined you are to overcome your personal challenges. Overcome the odds!

## Notes On Contributors

Awais Ul-Hassan is fourth year medical student at the University of Leeds. He is the eldest child in his family and was raised by single-parent mother in Yorkshire. Awais attended local state secondary school and sixth form and he successful attained a place to study medicine at the University of Leeds in 2016.

Salma Khanom is a qualified teacher in secondary education who runs a homework help centre for disadvantaged students in East London. She often supports applicants considering applying to medicine by encouraging them to volunteer by participating in her project.

Penelope Sucharitkul is a second year medical student at the University of Leeds. She is the only child from a single-parent family, and currently lives with her father who is a Factory Worker. Both parents have severe learning and mental health difficulties. She attended multiple primary schools, one state secondary school and later moved to a selective state grammar school in Essex.

Christopher Mark Jones is a Wellcome Trust Clinical Research Fellow and Honorary Specialty Registrar in Clinical Oncology at the University of Leeds and Leeds Cancer Centre. He completed all of his secondary and sixth form education in state schools that were in special measures, and is of the first generation in his family to attend university.

Mohammed Abdul Waduud is a British Heart Foundation (BHF) Research Fellow in Vascular Surgery working at the University of Leeds. He is the second eldest child of an immigrant Bangladeshi tailor and housewife. MAW attended a state secondary school in one of London’s most deprived Boroughs, Tower Hamlets, and later attained a scholarship to study at Westminster School. He graduated in Medicine from the University of Glasgow in 2013.

## Appendices

### Appendix

Appendix 1. *Competition ratio for Medical Schools in the United Kingdom.* Ratios highlight applicants to applicants interviewed, and applicants to overall places available. Empty cells indicate the absence of data (1).

**Table:**

Medical School	Applicants: Interview	Applicants: Places	Admissions Test Required
University of Aberdeen Medical School	2.4	11.6	UCAT
Anglia Ruskin University School of Medicine			UCAT
Aston University Medical School			UCAT
University of Birmingham Medical School MB ChB Medicine and Surgery	2	6	UCAT
Brighton and Sussex Medical School		10	BMAT
University of Bristol Medical School	3.9	10.5	UCAT
University of Buckingham Medical School	2.5	4.8	NONE
University of Cambridge School of Clinical Medicine		5	BMAT
Cardiff University School of Medicine	2	8	UCAT
University of Dundee School of Medicine	3	10	UCAT
University of East Anglia, Norwich Medical School	1.8	8.3	UCAT
University of Edinburgh Medical School			UCAT
University of Exeter Medical School			UCAT
University of Glasgow School of Medicine			UCAT
Hull York Medical School	1.7	7.9	UCAT
Imperial College London School of Medicine	3	7	BMAT
Keele University School of Medicine	2.5	10	UCAT
King’s College London School of Medical Education			UCAT
Lancaster University Medical School	1.7	8.2	BMAT
University of Leeds Medical School	2.9	7.8	BMAT
University of Leicester Medical School	2	8.7	UCAT
University of Liverpool School of Medicine	1.6	8.5	UCAT
University of Manchester School of Medicine	1.6	5.6	UCAT
Newcastle University Medical School	1.9	5.9	UCAT
University of Nottingham School of Medicine			UCAT
University of Nottingham Lincoln Medical School (University of Lincoln)			UCAT
University of Oxford Medical School	3.4	10.4	BMAT
Plymouth University Peninsula Medical School	4	8	UCAT
Queen Mary University, Barts and the London School of Medicine	3	7	UCAT
Queen’s University Belfast, Medical School	2	3	UCAT
University of Sheffield Medical School	1.7	6.6	UCAT
University of Southampton Faculty of Medicine	2	8	UCAT
University of St Andrews Medical School			UCAT
St George’s, University of London	1.8	6.9	UCAT
University of Sunderland School of Medicine			UCAT
University College London Medical School	3	7	BMAT
University of Central Lancashire Medical School		14	NONE

Appendix 2. *A Summary of Widening Participation Schemes Provided by Medical Schools in the UK.* Empty cells indicate the absence of data. (1)

**Table:**

	Medical School (33)	Widen Participation Scheme
1	University of Aberdeen Medical School	https://www.abdn.ac.uk/smmsn/undergraduate/medicine/widening-participation.php
2	Anglia Ruskin University School of Medicine	https://www.anglia.ac.uk/~/media/Files/fms/School%20of%20medicine/widening-access-to-medicine-questionnaire-september-2018.docx?la=en
3	Aston University Medical School	http://www.astonmedicalschool.com/pathway
4	University of Birmingham Medical School MB ChB Medicine and Surgery	https://www.birmingham.ac.uk/teachers/pupil-opportunities/post-16/routes-to-professions.aspx https://www.birmingham.ac.uk/teachers/pupil-opportunities/post-16/routes-to-professions.aspx
5	Brighton and Sussex Medical School	https://www.bsms.ac.uk/about/info-for-schools-teachers-parents/widening-participation-to-medicine.aspx https://www.bsms.ac.uk/undergraduate/applying-to-bsms/index.aspx
6	University of Bristol Medical School	http://www.bristol.ac.uk/study/undergraduate/entry-requirements-qualifications/contextual-offers/
7	University of Buckingham Medical School	
8	University of Cambridge School of Clinical Medicine	
9	Cardiff University School of Medicine	http://www.cardiff.ac.uk/study/undergraduate/applying/contextual-data
10	University of Dundee School of Medicine	https://www.dundee.ac.uk/study/widening-access/in-context/
11	University of East Anglia, Norwich Medical School	https://www.uea.ac.uk/study/info-for/teachers/post-16-information/medical-aspirations
12	University of Edinburgh Medical School	https://www.ed.ac.uk/studying/undergraduate/applying/selection/contextual-admissions
13	University of Exeter Medical School	
14	University of Glasgow School of Medicine	https://www.gla.ac.uk/study/wideningparticipation/
15	Hull York Medical School	https://www.hyms.ac.uk/medicine/applying-to-study-medicine/selection-procedure-in-full
16	Imperial College London School of Medicine	https://www.imperial.ac.uk/be-inspired/student-recruitment-and-outreach/schools-and-colleges/students/on-campus-activities/programmes/pathways-to-medicine/
17	Keele University School of Medicine	https://www.keele.ac.uk/medicine/mbchb5years/entryrouteshowtoapply
18	King’s College London School of Medical Education	Offer an Extended Medical Degree Programme: https://www.kcl.ac.uk/study/undergraduate/courses/extended-medical-degree-programme-mbbs
19	Lancaster University Medical School	http://www.lancaster.ac.uk/lms/about-us/widening-participation/
20	University of Leeds Medical School	http://www.leeds.ac.uk/info/128005/applying/33/alternative_admissions/2
21	University of Leicester Medical School	https://www2.le.ac.uk/offices/scs/activities/rop
22	University of Liverpool School of Medicine	https://www.liverpool.ac.uk/medicine/study-with-us/undergraduate/admissions-information/
23	University of Manchester School of Medicine	http://www.manchester.ac.uk/study/undergraduate/applications/after-you-apply/contextual-data/
24	Newcastle University Medical School	https://www.ncl.ac.uk/about/vision/partnerships/schools/
25	University of Nottingham School of Medicine	https://www.nottingham.ac.uk/medicine/study/medicine/widening-participation.aspx
26	University of Nottingham Lincoln Medical School (University of Lincoln)	https://www.nottingham.ac.uk/medicine/study/medicine/widening-participation.aspx
27	University of Oxford Medical School	http://www.ox.ac.uk/about/increasing-access/widening-access-and-participation
28	Plymouth University Peninsula Medical School	https://www.plymouth.ac.uk/your-university/about-us/university-structure/faculties/medicine-dentistry/widening-access
29	Queen Mary University, Barts and the London School of Medicine	http://www.qmul.ac.uk/undergraduate/teachers/wp/medicine-and-dentistry/index.html
30	Queen’s University Belfast, Medical School	https://www.qub.ac.uk/courses/undergraduate/pdf/howwechooseourstudents/Filetoupload,752882,en.pdf https://www.sheffield.ac.uk/medicine/prospective_ug/applying/wideningaccess
31	University of Sheffield Medical School	https://www.southampton.ac.uk/medicine/undergraduate/courses/bm6_a102.page
32	University of Southampton Faculty of Medicine	https://www.southampton.ac.uk/medicine/undergraduate/courses/bm6_a102.page
33	University of St Andrews Medical School	https://www.st-andrews.ac.uk/study/access/projects/reach/
34	St George’s, University of London	https://www.sgul.ac.uk/study/undergraduate/undergraduate-courses/medicine-mbbs/entry-criteria
35	University of Sunderland School of Medicine	
36	University College London Medical School	https://www.ucl.ac.uk/prospective-students/undergraduate/application/requirements/access-ucl https://www.ucl.ac.uk/target-medicine
37	University of Central Lancashire Medical School	https://www.uclan.ac.uk/courses/bachelor_medicine_bachelor_surgery.php

Appendix 3. *BMAT & UCAT Resources.* List of some free resources to help prepare for the UCAT and/or BMAT.

• https://www.ucat.ac.uk/ucat/practice-tests/
• https://www.themedicportal.com/teacher-services/teachers-guide-ukcat-and-bmat/
• https://themedicblog.co.uk/ucat-practice-questions/
• https://www.medentry.edu.au/resources
• https://medstart.com.au/free/
• https://www.medicmind.co.uk/resources.html
• https://6med.co.uk/bmat-resources/
• https://www.blackstonetutors.co.uk/free-bmat-resources.html
• https://www.bmatcrashcourse.com/free-resources/
• https://bmat.ninja/
• https://theukcatblog.com/bmat-sample-questions/
