# Supplementary material for: Twelve Tips for Applicants from a Disadvantaged Background Considering a Career in Medicine
Source: MedEdPublish (2016). 2019 Jun 28;8:144. [Version 1] doi: 10.15694/mep.2019.000144.1 (PMC10712481; doi:10.15694/mep.2019.000144.1)
Supplement: Peer review history

# Reviewer report: Reviewer response for version 1

**DOI:** 10.21956/mep.19869.r30028

**Published:** 2019-07-14

**Authors:** Helena Filipe

**Affiliations:** 1 Hospital of the Armed Forces/PL-EMGFA

This review has been migrated. The reviewer awarded 5 stars out of 5 Authors decided to tackle an important topic: advice on how to succeed applying to medical school despite a not so advantageous background. While recognizing that despite efforts to attain diversity, students eligible for programs to overcome these less favorable conditions they are still underrepresented. Furthermore there is no evidence showing that successful applicants will turn into underperforming students. Authors created a concise, clear and evidence informed background to anchor their article, which is a roadmap with practical advice and experience sharing. Their writing is clear, fluent and colloquial supporting authenticity to best reach the medical school candidates in tandem with the millennial generation mind set. They share practical experience that will turn useful for all applicants to Med School.The designation “difficult circumstances” is in itself compelling and brings, à priori relevance to what follows. The goal to reach out and assist for those in less favorable situations is noble and relevant. This is a valuable piece that certainly reflects the experience of the Authors in their particular context. However it can work as a showcase incentive to widen and attempt some worldwide agreement on these valuable tips. A new article can emerge from this one.Could imagine some kind of flowchart/mindmap to be visually attractive and the tips grabbed on a glimpse.

Reviewer Expertise:

NA

No decision status is available

# Reviewer report: Reviewer response for version 1

**DOI:** 10.21956/mep.19869.r30024

**Published:** 2019-07-12

**Authors:** Leila Niemi-Murola

**Affiliations:** 1 University of Helsinki

This review has been migrated. The reviewer awarded 4 stars out of 5 This is an interesting article giving 12 tips for applicants from disadvantaged backgrounds This is not just a British problem or one considering students from less prestigious schools. In Finland, a growing number of applicants from academic families are admitted as students to medical schools suggesting that socio-economical background is a meaningful background factor.Despite the heading, these 12 tips are as valid for any student considering application to medical school. Even offspring of physicians are not aware of the reality in healthcare and thus they are equally poorly prepared. The authors have sound recommendations about the importance of preparing for examinations and good advice how to boost ones self-esteem. Often admittance is the most difficult part of studying and, as such, one of the most empowering experiences in ones life.

Reviewer Expertise:

NA

No decision status is available

# Reviewer report: Reviewer response for version 1

**DOI:** 10.21956/mep.19869.r30029

**Published:** 2019-06-30

**Authors:** Johnny Lyon Maris

**Affiliations:** 1 Southampton GP Education Unit

This review has been migrated. The reviewer awarded 5 stars out of 5 Thankyou for submitting this article which is well researched for a UK audience and seems very personal to the authors. The approaches are a great start for a potential medical student who does not have easy access to traditional career advice. I can only re-inforce the statement that the candidate should not be put off by non-response, especially over work experience. Gaining contacts is key, however they will be different in every area so keep asking around. Well done with this article.

Reviewer Expertise:

NA

No decision status is available

# Reviewer report: Reviewer response for version 1

**DOI:** 10.21956/mep.19869.r30027

**Published:** 2019-06-29

**Authors:** Felix Silwimba

**Affiliations:** 1 University of Lusaka

This review has been migrated. The reviewer awarded 5 stars out of 5 I have enjoyed every bit of this article twelve tips for applicants from a disadvantaged background considering a career in medicine, even though I'm not UK based. If find the issues raised relevant to my situation living in a low-middle income country. I'm trying to inteprete the article in my contest. Take into consideration falling government support to University education in general and the rising of private medical and health education universities and colleges. then I position myself as an adviser to those aspiring to study medicine and health professional courses. Very useful and timely indeed.

Reviewer Expertise:

NA

No decision status is available

# Reviewer report: Reviewer response for version 1

**DOI:** 10.21956/mep.19869.r30025

**Published:** 2019-06-28

**Authors:** Lee HangFu

**Affiliations:** 1 Windsor University School of Medicine

This review has been migrated. The reviewer awarded 3 stars out of 5 This paper is very resourceful for the disadvantaged applicants. Unfortunately, the tips and resources offered here in this paper are very narrowly applicable only for those applying to UK medical schools.

Reviewer Expertise:

NA

No decision status is available

# Reviewer report: Reviewer response for version 1

**DOI:** 10.21956/mep.19869.r30026

**Published:** 2019-06-28

**Authors:** P Ravi Shankar

**Affiliations:** 1 American International Medical University

This review has been migrated. The reviewer awarded 4 stars out of 5 Thank you for the invitation to review this interesting paper. The article is well written. While many of the tips may be of benefit to international applicants most of the writing is focused toward applicants from the United Kingdom. I would suggest the authors modify the title of their paper to ‘Twelve Tips for Applicants from a Disadvantaged Background Considering a Career in Medicine in the United Kingdom’. There are a few spelling and language errors which can be corrected. It is commendable that the authors from disadvantaged backgrounds now studying at medical school offer advice to other applicants from a similar background. Some of the UK-centric activities like the Duke of Edinburgh award and the National Citizen Service may need to be explained for international readers.

Reviewer Expertise:

NA

No decision status is available
